# Can Abundance of Protists Be Inferred from Sequence Data: A Case Study of Foraminifera

**DOI:** 10.1371/journal.pone.0056739

**Published:** 2013-02-19

**Authors:** Alexandra A-T. Weber, Jan Pawlowski

**Affiliations:** 1 Department of Genetic and Evolution, University of Geneva, Sciences III, Genève, Switzerland; 2 Aix-Marseille Université, IMBE UMR CNRS 7263, Institut Méditerranéen de Biodiversité et d'Ecologie marine et continentale, Station Marine d'Endoume, Marseille, France; Université Paris Sud, France

## Abstract

Protists are key players in microbial communities, yet our understanding of their role in ecosystem functioning is seriously impeded by difficulties in identification of protistan species and their quantification. Current microscopy-based methods used for determining the abundance of protists are tedious and often show a low taxonomic resolution. Recent development of next-generation sequencing technologies offered a very powerful tool for studying the richness of protistan communities. Still, the relationship between abundance of species and number of sequences remains subjected to various technical and biological biases. Here, we test the impact of some of these biological biases on sequence abundance of SSU rRNA gene in foraminifera. First, we quantified the rDNA copy number and rRNA expression level of three species of foraminifera by qPCR. Then, we prepared five mock communities with these species, two in equal proportions and three with one species ten times more abundant. The libraries of rDNA and cDNA of the mock communities were constructed, Sanger sequenced and the sequence abundance was calculated. The initial species proportions were compared to the raw sequence proportions as well as to the sequence abundance normalized by rDNA copy number and rRNA expression level per species. Our results showed that without normalization, all sequence data differed significantly from the initial proportions. After normalization, the congruence between the number of sequences and number of specimens was much better. We conclude that without normalization, species abundance determination based on sequence data was not possible because of the effect of biological biases. Nevertheless, by taking into account the variation of rDNA copy number and rRNA expression level we were able to infer species abundance, suggesting that our approach can be successful in controlled conditions.

## Introduction

Determining the abundance of protists is essential for understanding ecosystem functioning, seasonal communities' turnover and finding key-species in ecosystems. Furthermore, several groups of protists are commonly used as bioindicators for environment quality assessment [1]–[3].

So far, determination of protist abundance relied mainly on direct microscopic counting, quantitative PCR [4] and flow cytometry (FCM) [5], [6]. Standard microscope observation, beside the fact that it is time-consuming, can underestimate the real diversity as cryptic species are common in protists. Concerning flow cytometry, the separation of organisms is based on fluorescence response and on size and shape of the organisms. It is useful to determine the broad functional groups that are present in an environmental sample, but its taxonomic resolution is low. Furthermore, this method is only suitable for quantification of phototrophic organisms in water samples. Quantitative PCR can accurately estimate the copy number of a targeted gene present in a sample [7], but it is difficult to link qPCR results with abundance of protists if the gene copy number per species is unknown. Consequently, scientists have to make compromises to choose between analysis speed and taxonomic resolution when studying protist abundance.

Recent development of next-generation sequencing (NGS) methods prompted a new interest in inferring relative abundance of species from NGS-generated sequence data. This problem has been approached in bacteria [8]–[10] but left unsettled in protists. Some authors used 454 pyrosequencing data to infer seasonal species turnover in protists, but did not examine the correlation between relative abundance of species and sequence read proportions [11]. When this correlation was tested in fungal mock communities [12], the authors found a difference of one order of magnitude in sequence read abundance between the most and least abundant species, although the cells were in the same initial concentration. The authors pointed up the experimental biases (DNA extraction, PCR, sequencing) but also the fact that they did not know the rDNA copy number in the species they used, which could lead to important bias in estimation of species proportion. This bias was indeed observed by some authors in *in silico* analysis of a hypothetical microbial community [13].

We choose foraminifera as a model group of protists to examine the importance of biological biases, such as rDNA copy number or rRNA expression level variation in sequence quantification. Foraminifera are widely distributed in various marine habitats, with forms adapted to benthic as well as planktonic way of life. They are extremely diverse and present in virtually every marine ecosystem, from the coastal waters to the deep-sea, but also in water column and brackish water habitats. Foraminifera are commonly used in pollution monitoring, as they are very sensitive to environmental physico-chemical variations [14]. Their response to pollution reveals changes in assemblages and test deformation [2]. They are also widely used to assess the impact of anthropogenic activities [15]–[17].

In addition to their importance as bioindicators, the foraminifera were chosen for our study because they are relatively large and it is easy to isolate them one by one to prepare mock communities. However, foraminifera possess some particularities that hamper their use in experimental studies. First of all, they are difficult to maintain in laboratory culture and only few species regularly reproduce in culture conditions. Moreover, no complete genome of foraminifera is currently available, so the exact copy number of rRNA genes remains unknown. Furthermore, their life cycle comprises an alternation of generation between uninucleate haploid gamont and multinucleate diploid agamont [18]. Recently, some authors [19] showed that a polyploid phase exists in life cycle of some foraminiferal species. Besides, changes in number of nuclei during the life cycle have also been reported [20]. Taking this into consideration, the rDNA copy number in foraminifera can vary between species but also within species.

To overcome this problem, a quantitative PCR method was used to infer rDNA copy number as well as rRNA expression level in three species of cultured foraminifera. We chose a fragment of small subunit (SSU) rRNA genes that is commonly used as barcode of foraminiferal species [21].

After having estimated the SSU rDNA copy number and SSU rRNA expression level for each species, respective normalization factors were calculated. The factors corresponded to the differences in SSU rDNA copy number (Copy Number Factor) and in SSU rRNA expression level (Expression Level Factor) among species. Then, mock communities of these three species were prepared, with cells in equal quantities as well as with one species in majority (ten times more abundant). The SSU rDNA fragment amplified with specific foraminiferal primers was cloned and Sanger sequenced. The sequences were counted, and their proportions were compared to initial cell proportions, to see if sequence data are relevant for abundance determination. Finally, to infer if the SSU rDNA copy number or SSU rRNA expression level influences significantly the sequences results, the number of sequences was divided by the CNF and ELF found by qPCR, and the proportions of sequences newly calculated was again compared to the initial proportions of cells.

## Materials and Methods

### Cultures

Three species of Foraminifera were used for this experiment, namely *Allogromia laticollaris* strain CSH (Lee, 1969), *Rosalina* sp. and *Bolivina variabilis* (d'Orbigny, 1843). *Allogromia laticollaris* CSH was isolated by Dr. John Lee from Cold Spring Harbour (New York State, USA) in 1969 and its culture was send to Geneva by courtesy of Laura Parfrey. *Rosalina* specimens were collected at the coast of Langeoog island (North Sea, Germany) by researchers of Tübingen University and were maintained in culture since 1984. *Bolivina variabilis* specimens were collected at the coast of Porquerolles Island (Mediterranean Sea, France) by JP in September 2008. The Foraminifera were maintained in glass culture dishes stored in an incubator (Aqualytic) at temperature maintained at 21°C. Once a week, filtered seawater was added to the cultures, with Erdschreiber medium [22] and heat-killed *Chlorella* used as a food. The three species were observed to reproduce asexually by schizogony. No specific permits were required for collecting the foraminiferal species used in this study.

### DAPI staining

Ten cells of each species were stained with DAPI (4′,6-diamidino-2-phenylindole) [23] to reveal their nuclei. Cells were first cleaned from diverse organisms attached to their test with a brush, and picked up from the Petri dish with a 20 µl micropipette. *Rosalina* and *Bolivina* specimens were fixed in 200 µl formaldehyde 4% overnight, washed with distilled water and incubated with DAPI 0.001% in water, for one hour at 37°C in the dark. *Allogromia* specimens were fixed in 200 µl formaldehyde and 0.5% Triton X 100 one hour on ice, washed with distilled water and incubated with DAPI 0.001% in water, for one hour at 37°C in the dark. Cells were observed and photomicrographed under UV light (excitation filter EX330-380 and barrier filter BA420) and transmitted light with a Nikon Eclipse E200 epifluorescent microscope and Nikon digital camera DXM 1200.

### DNA and RNA extractions

A total of 200 specimens of *Allogromia*, *Rosalina* and *Bolivina* were picked for DNA and RNA extractions. Each extraction was performed with a pool of 10 individuals per species. DNA was extracted with DNeasy Plant Mini Kit (Qiagen) following the manufacturer's instructions and eluted in a final volume of 100 µl. All DNA extractions were stored at −20°C. RNA was extracted with the Nucleospin RNA XS Kit (Macherey-Nagel) following the manufacturer's instructions and eluted in a final volume of 10 µl. cDNA was generated with the foraminiferal specific primer s17 (Table S1) using the iScript Select cDNA Synthesis Kit (BioRad) following the manufacturer's instructions.

### Quantitative PCR

For quantifying DNA and cDNA assays, a standard curve was constructed with the sequence of *Rosalina* (culture species). Thirty cells of *Rosalina* were pooled and DNA was extracted with DNeasy Plant Mini Kit (Qiagen) following the manufacturer's instructions. A SSU rDNA fragment (1081 bp) was amplified with primers (14F3 and sBN, Table S1) in standard PCR conditions. PCR products were then cloned with TOPO TA Cloning Kit (Invitrogen) following the manufacturer's instructions. One clone having a *Rosalina* insert was grown overnight in LB medium and used as standard for qPCR. Plasmid extraction was then performed with peqGOLD Plasmid Mini Kit (PeqLab) following the manufacturer's instructions. Plasmid concentration was first roughly measured with Nanodrop ND-1000 spectrophotometer (Nanodrop Technologies) and the plasmid extraction was stored at −20°C. The number of rDNA molecules in the plasmid extract was calculated using the formula from Rodriguez-Martinez [24].

As the quantification with Nanodrop spectrophotometer is less precise than fluorometry quantification, a new fluorometrically measure was done for each qPCR plate, to optimize the plasmid concentration measurement. As the plasmid stock concentration was estimated at 10^10^ molecules/µl with Nanodrop quantification, serial dilution were performed on this basis; dilutions for cDNA samples quantitation ranging from 10^8^ to 10^4^ copies/µl, and dilutions for DNA samples quantitation ranging from 10^6^ to 10^2^ copies/µl. 2 µl of plasmid stock were added to 198 µl of water and this first dilution was quantified with the Qubit 2.0 fluorometer (Invitrogen). Then, serial dilutions were performed from this quantified dilution until reaching the desired dilutions above-mentioned.

The region chosen for qPCR assays was the second hypervariable region of the SSU rDNA gene (41/f) [21] of the length of 170–194 bp in the three examined species. Reactions were performed using hard-shell thin wall 96-well skirted PCR plates (Bio-Rad) and sealed with optical strips (Bio-Rad). In a final volume of 20 µl using SsoFast EvaGreen Supermix Kit (Bio-Rad), each reaction contained 10 µl of SsoFast EvaGreen Supermix (2× reaction buffer with dNTPs, Sso7d-fusion polymerase, MgCl_2_, EvaGreen dye and stabilizers), 500 nM of each primer (s15R1 and s17) (Table S1) and 3, 6 or 7 µl of Nuclease free water for 5 µl of DNA sample, 2 µl of cDNA sample and 1 µl of DNA standard, respectively. All reactions were performed in triplicates with the CFX96 Real-Time PCR Detection System (Bio-Rad) with the following program: An initial enzyme activation step of 2 min at 98°C followed by 40 cycles of 5 s of denaturation at 98°C, 5 s of annealing-extension at 56°C and data collection at the end of annealing-extension step. Finally, the dissociation curve of the amplicons was measured after the last qPCR cycle, from 56°C to 95°C by increasing 0.5°C every 5 s. This measure was useful to detect primer-dimer or DNA contaminations as EvaGreen dye binds every double-strand DNA without specificity. The presence of a unique peak in the melting curve meant a successful amplification.

### Normalization factors

DNA assays were performed with 5 µl of DNA extraction, and elution volume of the DNA extractions was 100 µl. Consequently, to infer the number of SSU rDNA copies per cell per species, the qPCR result for one extraction was multiplied by (20/10). The mean SSU rDNA copy number per cell per species was then calculated for each result of the two qPCR plates. In the same way, raw RNA data were used to determine the mean SSU rRNA expression level of ten specimens per species. Normalization factors between species, CNF and ELF, were then found by dividing the quantification results of the two species that showed a greater SSU copy number or expression level by the quantification result of the smallest one. The CNF and ELF were then used to normalize the sequencing results, by dividing the number of sequences found for each species by the corresponding CNF and ELF. Thus, the SSU rDNA copy number and SSU rRNA expression level could artificially be at the same level between *Allogromia*, *Rosalina* and *Bolivina*.

### Sanger sequencing of mock communities

Five mock communities of *Allogromia*, *Rosalina* and *Bolivina* were prepared in different proportions of cells for DNA and cDNA sequencing (Table S2). Two mock communities were done with the cells in equal proportions: with three cells of each species (mix 3) and with ten cells of each species (mix 10). Three mock communities were done with one species ten times more abundant (mix *Allogromia*, mix *Rosalina*, mix *Bolivina*; thirty cells for the most abundant species with three cells for the two other species) (Table S2). Five DNA and five RNA extractions were performed with the kits previously quoted. Three PCR replicates were performed on the DNA extractions and one PCR was performed on each cDNA sample with primers s14F1 and s17 (Table S1) in standard PCR conditions (fragment length 322–360 bp). The PCR products were then cloned as described above and sequenced using Big Dye Terminator v3.1 Cycle Sequencing Kit (Applied Biosystems) and an automatic sequencer ABI Prism 3130XL (Applied Biosystems).

### Sequence analysis, Chi^2^ test and normalization

Sequences were manually edited with the software 4Peaks, then aligned in the program Seaview [25] with MUSCLE v3.8.31 algorithm [26] and manually improved. After removing the chimeras (about 1%), sequences were blasted against the foraminiferal database of the laboratory, and identified as *Allogromia*, *Rosalina* or *Bolivina*. For each mock community, sequences of the three species were counted and the proportion of each species was calculated. A Chi^2^ goodness of fit Test [27] was performed on these proportions, by comparing the initial proportions of the cells in each mix (expected proportions) with the proportions of the sequences found after sequencing (observed proportions), with the R software. The number of sequences per species in each mock community was then normalized by the CNF and ELF found in qPCR experiment, to remove the bias due to unequal SSU rDNA copy number or SSU rRNA expression level. In the same way, the normalized proportions were compared to initial cell proportions with a Chi^2^ goodness of fit Test.

### RFLP Analysis

In addition to sequencing, we developed a RFLP protocol to rapidly recognize the foraminiferal species present in the mock communities. Two restriction enzymes were used simultaneously, namely *Dra I* (cutting site: TTT/AAA) and *Cfo I* (cutting site GCG/C) (Boeringer-Mannheim) that generate different fragment lengths specific of each species (Table S3). Restriction simulations were done with the program EnzymeX, and verified with PCR products whose sequence was known. A total of 192 colonies were picked in the five Petri dishes of cDNA mock communities and the results were added to the number of sequences found by sequencing. Furthermore, the sequences of the two PCR replicates performed on the DNA extractions were identified by RFLP analysis only. A total of 1749 colonies were picked in the ten Petri dishes of DNA mock communities (two additional replicates) and PCR were performed as previously described, with vector primers. Digestion by *Cfo I* and *Dra I* was directly performed on the PCR products. 1 U of each of restriction enzymes, 2.5 µl of Buffer L (Boeringer-Mannheim), and 4.5 µl of water were added to 12.5 µl of PCR product. Digestion was performed 90 min at 37°C. Restriction patterns were differentiated by migrating the digestion products on a 2% agarose gel (TBE 0.5X) stained with SYBR safe and detected under UV light. As the TA cloning technique is non-directional, there were two types of fragments for each species depending on the cloning sense of the insert (Table S3)

## Results

### Species morphology and nuclear apparatus

**Figure 1:**
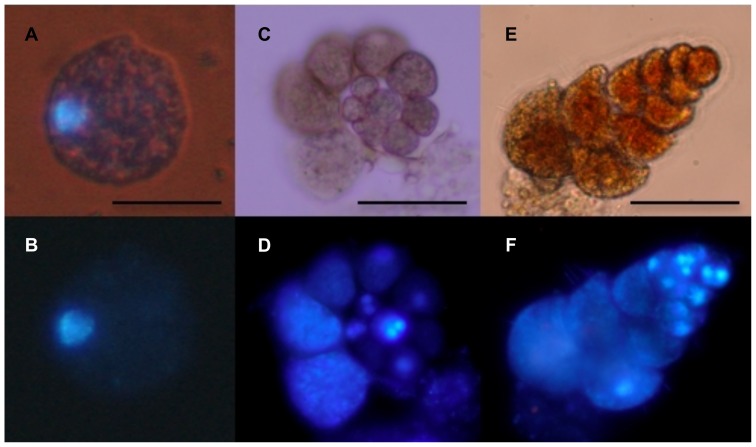
Micrographs of living foraminifera. The photos of *Allogromia laticollaris* (A, B), *Rosalina* sp. (C, D) and *Bolivina variabilis* (E, F) under transmitted and UV light, showing fixed specimens with DAPI stained nuclei. Scale bars are 50 µm.

*Allogromia laticollaris* is a monothalamous (single-chambered) foraminifer that possesses an organic spherical theca (Figure 1A). The largest specimens measure up to 300 µm, but those used in this study measured in average 150 to 200 µm. The DAPI staining showed that all examined specimens possessed one big nucleus (Figure 1B). However, specimens with several small nuclei representing different life-cycle stage were observed by others [20].

*Rosalina* sp. is a polythalamous (multi-chambered) foraminifer that possesses a calcareous test. The test is composed of the proloculus (first central chamber) and several successive chambers forming a spiral coil (Figure 1C). Its size reaches up to 500 µm, but smaller specimens of 150 to 200 µm were used in this study. *Rosalina* sp. is multinucleated with two bright nuclei present in the proloculus and several nuclei visible in the next chambers (Figure 1D). Due to the thickness of *Rosalina* wall, DAPI staining was rarely successful and the nuclear apparatus of this species was difficult to characterize.

*Bolivina variabilis* is a polythalamous species with a biserial (built with two series of successive chambers) calcareous test. *Bolivina* measures up to 200 µm, but in average the specimens used in this study measured about 100 µm (Figure 1E). Contrary to *Rosalina*, DAPI staining was always successful and all the observed specimens were multinucleated (Figure 1F). Roughly estimated, the number of nuclei varied between 5 and 12, but the observation conditions were not always optimal.

### Estimation of CNF and ELF

**Figure 2:**
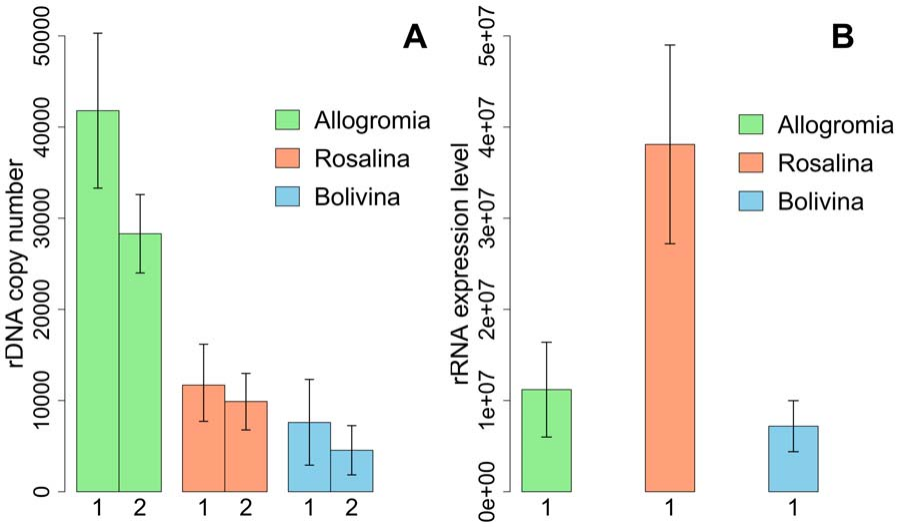
Results of qPCR assays. (A) SSU rDNA copy number estimation per cell per species inferred from qPCR data, based on two replicate measurements. (B) SSU rRNA expression level per cell per species inferred from qPCR data based on one replicate measurement. Error bars are standard deviation.

After optimization of qPCR assays, two qPCR plates were successfully performed for SSU rDNA copy number estimation. A total of seven extractions of ten cells were quantified for each species. *Allogromia* displayed a significantly higher SSU rDNA copy number than *Bolivina* and *Rosalina* in the two qPCR plates results (Figure 2A). *Allogromia laticollaris* CSH seemed to have in average between 30 000 and 40 000 SSU rDNA copies, while *Rosalina sp.* seemed to possess about 10 000 SSU rDNA copies and *Bolivina variabilis* between 5 000 and 10 000 SSU rDNA copies (Table S4). The CNFs (Table S4) used for normalization of sequence data were calculated based on *Bolivina* quantification results of the first qPCR plate.

The quantification of SSU rRNA expression level was based on one qPCR plate only, as the second plate failed to give satisfactory results. Interestingly, *Rosalina* displayed a significantly higher SSU rRNA expression level than *Allogromia* and *Bolivina* (Figure 2B). There was no correlation between SSU rDNA copy number and SSU rRNA expression level, as it was shown that *Allogromia* possessed the highest SSU rDNA copy number, and *Rosalina* the highest SSU rRNA expression level. The ELFs (Table S5) used for normalization of sequence data were calculated based on *Bolivina* quantification results, as it showed the smallest expression level (Figure 2B).

### Analysis of mock communities

**Figure 3:**
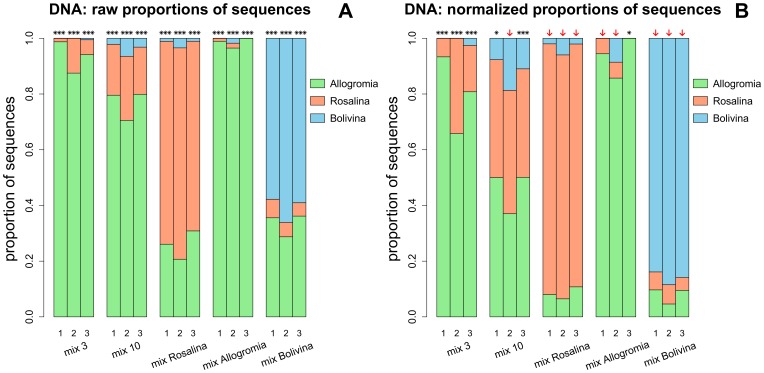
Proportion of rDNA sequences of *Allogromia, Rosalina* and *Bolivina* found in the five mock communities. (A) Observed proportions of rDNA sequences without normalization; (B) Observed proportions of SSU rDNA sequences after normalization with the CNF (Copy Number Factor, see text for details). Raw and normalized proportions were used for Chi^2^ goodness of fit test (df = 2; α = 0.05) ***: significant at 0.001; **: significant at 0.01; *: significant at 0.05; arrow: not significant, meaning that observed proportions did not differ significantly from expected proportions. Numbers 1, 2 and 3 correspond to the replicate number. Mix 3 - with three cells of each species; mix 10 - with ten cells of each species; mix Allogromia – with thirty cells of *Allogromia* and three cells of *Rosalina* and *Bolivina*; mix Rosalina – with thirty cells of *Rosalina* and three cells of *Allogromia* and *Bolivina*; mix Bolivina – with thirty cells of *Bolivina* and three cells of *Allogromia* and *Rosalina*.

DNA of five mock communities (mix 3, mix 10, mix *Allogromia*, mix *Rosalina* and mix *Bolivina*) was extracted and a fragment of SSU rDNA was amplified, cloned and sequenced or analyzed by RFLP. The number of rDNA sequences analyzed varied between 79 and 94 in the replicate 1, between 139 and 174 in the replicate 2 and between 187 and 189 in the replicate 3 (Table S6). In the three DNA replicates, the observed proportions of sequences of each mock community differed significantly from expected proportions (Figure 3A). After normalization of sequence data with the CNF, the observed proportions of sequences did not differ significantly from expected proportions in 9 out of 15 cases (Figure 3B). Thus, we were able to infer species abundance based on normalized sequence data in almost all experimental conditions, except for the mix 3 (all replicates), the mix 10 (2 replicates) and the mix *Allogromia* (one replicate). In the later case, only sequences of *Allogromia* were found in the 187 sequences analyzed (Table S6).

**Figure 4:**
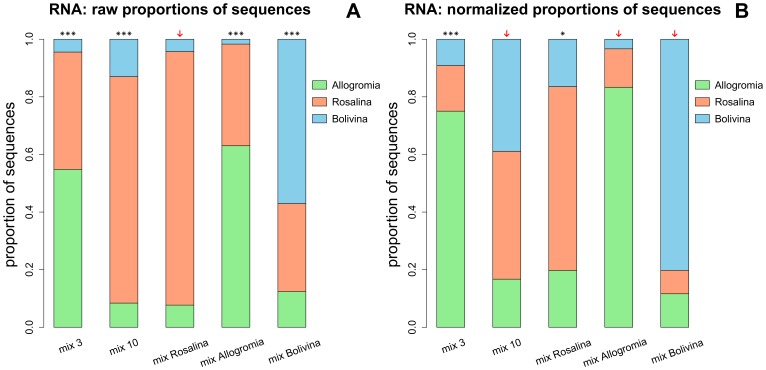
Proportions of cDNA sequences of *Allogromia*, *Rosalina*, and *Bolivina* found in the five mock communities. (A) Observed proportions of cDNA sequences without normalization; (B) Observed proportions of cDNA sequences after normalization with the ELF (Expression Level Factor, see text for details). Raw and normalized proportions were used for Chi^2^ goodness of fit test (df = 2; α = 0.05). ***: significant at 0.001; **: significant at 0.01; *: significant at 0.05; arrow: not significant, meaning that observed proportions did not differ significantly from expected proportions. Mix 3 - with three cells of each species; mix 10 - with ten cells of each species; mix Allogromia – with thirty cells of *Allogromia* and three cells of *Rosalina* and *Bolivina*; mix Rosalina – with thirty cells of *Rosalina* and three cells of *Allogromia* and *Bolivina*; mix Bolivina – with thirty cells of *Bolivina* and three cells of *Allogromia* and *Rosalina*.

The analysis of rRNA expression level was done in the same way as in the case of rDNA, but without replicates. The number of cDNA sequences analyzed varied between 117 and 135 (Table S7). The results showed that without normalization, sequence proportions of all mixes, except mix *Rosalina* significantly differed from expected proportions (Figure 4A). After normalization by the ELF, the proportions of sequences of the mixes 10, *Allogromia* and *Bolivina* did not differ significantly from expected proportions, indicating that normalization was successful to infer species abundance based on sequence abundance (Figure 4B). However, the normalization did not work in the case of *Rosalina* mix, which curiously was significantly different from expected proportion. Like in the case of rDNA study, the normalization had no effect on the mix 3 dominated by high proportion of *Allogromia* sequences, particularly in the raw sequence data.

## Discussion

### SSU rDNA copy number in Foraminifera

Before the present study, nothing was known about the number of SSU rDNA copies in Foraminifera. It is widely accepted that SSU rDNA copy number varies considerably among eukaryotic species [28] but there are few systematic studies that examine the range of these variations in unicellular eukaryotes. In our study, we estimated the rDNA copy number between 10 000 and 30 000 for the three studied foraminiferal species. This number may appear extremely elevated, in comparison to 1–15 copies found in bacteria [29], 30 copies found in marine stramenopiles MAST4 [24] and around 150 copies in *Saccharomyces cerevisiae* [30]. Yet, much higher numbers were reported in ciliates, ranging from 3415 in parasitic species *Cryptocaryon irritans* [31] to 160,000 copies in *Vorticella* sp. [32] and up to 200,000 in macronucleus of *Stylonychia lemnae* [33]. It has been shown that the rRNA gene copy number correlates with genome size [28], cell length [4] and biovolume [34]. Zhu et al. [4] predict about 10,000 rDNA copies for a 100 µm cell, which is in agreement with our data given that the examined foraminiferal cells range in size between 100 and 200 µm. Thus, the estimated rDNA copy number seems to be in the right order of magnitude.

It is interesting to notice that the rDNA copy number seems not related to the number of nuclei in foraminifera. The uninuclear *Allogromia* shows up to five times as many rDNA copies as multinucleate *Rosalina* and *Bolivina*. This could be explained by high polyploidy observed in the uninucleate stage of *Allogromia* Agamont I [20]. It cannot be excluded that not all specimens of *Allogromia* were uninucleated and that some of them belong to the multinucleate Agamont II stage. On the other hand, *Rosalina* and *Bolivina* belong to the order of Rotaliida, which is distantly related to the allogromiids. Although polyploidy was suggested in the case of some large rotaliids of the family Nummulitidae [35] it is possible that the small rotaliids, such as *Rosalina* and *Bolivina* do not undergo polyploidy and have relatively stable number of rDNA copies.

### Impact of rDNA copy number, rRNA expression level and possible biases

As expected, the rDNA copy number and rRNA expression level have strong impact on proportion of sequences in rDNA and cDNA libraries. Our results showed that without normalization, it was not possible to correctly determine abundance of species based on proportions of sequences. In 39 cases out of 40 the observed proportions differed significantly from the expected proportions. By using normalization factors that removed artificially the “excess” of sequences due to a high rDNA copy number or high rRNA expression level, we corrected the differences between observed and expected proportion of sequences and we obtained positive results for many mock communities with species in equal proportions and ten times more abundant.

Nevertheless, in some cases the normalization did not work, especially for the DNA and RNA data of mix 3. This could by explained by the very low number of cells used in those mock communities (9 cells). In this case, the experimental biases [36] could have much more influence on the results. In addition, working with so few cells raised drastically the “individual” effect due to inter-individual variations, as it is known that life cycle of foraminifera includes changes in ploidy and number of nuclei [19], [20]. Indeed, we could see that in the mix 3 of cDNA data, *Allogromia* sequences were the most abundant although we expected *Rosalina* sequences to be the most abundant, as it displayed the highest rRNA expression level. In this mix, the three *Allogromia* cells were probably in much more active stage than *Rosalina* cells, which can be explained by lack of synchronization of culturing stages of different foraminiferal species. This demonstrated that there was a significant bias when using so few cells for a mock community and we thus predict that the estimation of species abundance may be difficult in the case of rare species.

We also observed the presence of a slight PCR bias [36], as the results of the three DNA replicates were different even though the PCRs were done with the same DNA extractions. This could be explained by a possible preferential amplification of *Allogromia* rDNA, as it was the most abundant and it displayed the highest rDNA copy number. This problem could be avoided by using more cells in the mock communities and a sequencing technique that displays higher sequencing depth. Indeed in some cases, we also observed a sampling bias due to a low sequencing depth of cloning technique. We think that the use of next generation sequencing will drastically reduce the occurrence of sampling bias, but it remains to be tested if next generation sequencing does not bring additional biases.

It was surprising to observe a strong difference in rRNA expression level between *Allogromia* and *Rosalina*. Knowing that both species have similar cell sizes and regularly reproduced, we could expect them to display similar rRNA expression levels. Yet, rRNA expression level of *Rosalina* was almost four times greater than the expression level of *Allogromia*. Rather than a true smaller rRNA expression level, we suppose that there is a problem with the RNA extraction in the case of *Allogromia*. Indeed, several attempts to extract high quantities of total RNA of *Allogromia* failed (Roberto Sierra, personal comment). This points out the importance of choosing an adequate method for RNA and DNA extractions.

### Can abundance be inferred from the sequence data?

Despite of the presence of some biases, the general results of our study are very promising. Most of all, the impact of experimental biases, such as DNA/RNA extraction method, PCR conditions or primers selectivity seemed to be relatively limited. These biases are considered by some authors [37]–[40] as main factors compromising the quantitative approach of environmental DNA surveys. Although we do not really assess the importance of experimental conditions, our study clearly point to the initial amount of material, namely SSU rDNA copy number or SSU rRNA expression level, as the main factors responsible for the lack of correlation between the abundance of species and their sequences. Indeed, when looking at raw sequence data of mock communities with one species in majority, we could observe that the majority of sequences always corresponded to the initial most abundant species (Figure 3A, 4A). This means that even without normalization, the most abundant species could be found in sequence data, at least if the normalization factor was smaller than the initial proportion difference.

Foraminifera are particularly prone to biases in inferring the abundance from rDNA sequences due to alternation of generation, variation in ploidy, and variation in number of nuclei. Nevertheless, despite the high variation of rDNA copy number, we were able to infer species abundance based on sequence data in almost all foraminiferal mock communities. This result showed that the normalization technique worked even in highly variable system such as in foraminifera. Thus, working with other protists that display a more stable rDNA copy number could lead more easily to an accurate cell quantification based on sequence data. Indeed, Rodriguez-Martinez and colleagues [24] showed that there was a good correlation between the number of rDNA molecules and number of cells in MAST-4 group. This demonstrates that rDNA copy number can be stable within a smaller taxonomic group, confirmed also by our observations in the case of Rotaliida. We are aware that our approach requires a previous characterization of the species rRNA genes, so it cannot be applied to the unknown biodiversity. Yet, in a biomonitoring perspective where the bioindicator species are known and their number is limited, the previous estimation of rDNA copy number to work around this biological bias could be feasible and an accurate cell quantification based on sequence data could be realizable. Moreover, if the correlation between the number of rDNA copies and biomass is confirmed in foraminifera, as in some other protists [34], the sequence data could also provide a proxy for the activity of foraminiferal species.

Concerning the use of rRNA sequence data for describing abundance of protists, we recommend not using those data for inferring species abundance. In addition to the fact that the density of ribosomes can change in different life stages, the rRNA expression level can vary depending to various environmental factors that may change in time. An ELF calculated for one species in a certain period could thus be unusable at a different time of the year. It will be more appropriated to use the rRNA sequence data without referring to the species abundance but rather as a new ecological descriptor showing the active part of the community. In this way, the analysis of environmental rDNA and rRNA sequence data could give us complementary views on ecology of protist communities, the first one by considering the biodiversity and the abundance of protists and the second by considering the active part of the studied community.

## Supporting Information

Table S1 **PCR primers used for this study.**
(DOC)

Table S2 **Number of cells of each species used for the experimental mixes.**
(DOC)

Table S3 **Restriction fragments generated after digestion of PCR products of** ***Allogromia*** **,** ***Rosalina*** **and** ***Bolivina*** **by Dra I and Cfo I restriction enzymes.**
(DOC)

Table S4 **SSU rDNA copy number estimation for** ***Allogromia*** **,** ***Rosalina*** **and** ***Bolivina*** **inferred from qPCR data and corresponding copy number factors relatively to** ***Bolivina*** **results, used for normalization of the sequence data.**
(DOC)

Table S5 **SSU rRNA expression level for** ***Allogromia*** **,** ***Rosalina*** **and** ***Bolivina*** **inferred from qPCR data and corresponding Expression Level Factors (ELF) relatively to** ***Bolivina*** **results, used for normalization of the sequence data.**
(DOC)

Table S6 **Number of rDNA sequences of** ***Allogromia*** **,** ***Rosalina*** **,** ***Bolivina*** **found after cloning and sequencing of five PCR products of the mixes of species (Replicate 1), RFLP analysis of five PCR products of the mixes of species (Replicates 2 and 3).**
(DOC)

Table S7 **Number of cDNA sequences of** ***Allogromia*** **,** ***Rosalina*** **,** ***Bolivina*** **found after cloning and sequencing of PCR products of the five mixes of species and RFLP analysis.**
(DOC)
