# Supplementary material for: Can Abundance of Protists Be Inferred from Sequence Data: A Case Study of Foraminifera
Source: PLoS One. 2013 Feb 19;8(2):e56739. doi: 10.1371/journal.pone.0056739 (PMC3576339; doi:10.1371/journal.pone.0056739)
Supplement: Table S1 — PCR primers used for this study. (DOC) [file pone.0056739.s001.doc]

Table S1: PCR primers used for this study.

| Primer | Sequence (5’ 🡪 3’) | Direction | Length | Specificity | Tm (°C) |
| --- | --- | --- | --- | --- | --- |
| s17 | CGG TCA CGT TCG TTG C | reverse | 16 | Foraminifera | 57.8 |
| s15R1 | CAT GGC CGT TCT TAG TT(CG) GTG | forward | 21 | Eukaryote | 59.6 |
| s14F3 | ACG CA(AC) GTG TGA AAC TTG | forward | 18 | Foraminifera | 56.2 |
| sBN | TGC CTT GTT CGA CTT CTC | reverse | 18 | Foraminifera | 55.8 |
| s14F1 | AAG GGC ACC ACA AGA ACG C | forward | 19 | Foraminifera | 62.2 |
