# Supplementary material for: Can Abundance of Protists Be Inferred from Sequence Data: A Case Study of Foraminifera
Source: PLoS One. 2013 Feb 19;8(2):e56739. doi: 10.1371/journal.pone.0056739 (PMC3576339; doi:10.1371/journal.pone.0056739)
Supplement: Table S2 — Number of cells of each species used for the experimental mixes. (DOC) [file pone.0056739.s002.doc]

Table S2: Number of cells of each species used for the experimental mixes.

|  | *Allogromia* | *Rosalina* | *Bolivina* | Total |
| --- | --- | --- | --- | --- |
| Mix 3 | 3 | 3 | 3 | 9 |
| Mix 10 | 10 | 10 | 10 | 30 |
| Mix *Allogromia* | 30 | 3 | 3 | 36 |
| Mix *Rosalina* | 3 | 30 | 3 | 36 |
| Mix *Bolivina* | 3 | 3 | 30 | 36 |
