# Supplementary material for: Can Abundance of Protists Be Inferred from Sequence Data: A Case Study of Foraminifera
Source: PLoS One. 2013 Feb 19;8(2):e56739. doi: 10.1371/journal.pone.0056739 (PMC3576339; doi:10.1371/journal.pone.0056739)
Supplement: Table S3 — Restriction fragments generated after digestion of PCR products of Allogromia , Rosalina and Bolivina by Dra I and Cfo I restriction enzymes. (DOC) [file pone.0056739.s003.doc]

Table S3: Restriction fragments generated after digestion of PCR products of *Allogromia, Rosalina* and *Bolivina* by Dra I and Cfo I restriction enzymes.

|  | Entire fragment (bp) | Digested fragments ^1^ 1 (bp) | Digested fragments 2 (bp) |
| --- | --- | --- | --- |
| *Allogromia* | 632 | 209, 166, 137, 120 | 256, 209, 120, 47 |
| *Rosalina* | 628 | 195, 145, 120, 118, 50 | 235, 145, 120, 78, 50 |
| *Bolivina* (A)^2^ | 601 | 292, 166, 120, 23 | 409, 120, 49, 23 |
| *Bolivina* (B) | 613 | 327, 166, 120 | 444, 120, 49 |

^1^As the cloning technique is TA (no directional cloning) two types (1 and 2) of fragments appear depending on the cloning sense of the insert.

^2^ Different *Bolivina* sequence types (A and B) were found. Single-cell PCRs and cloning were performed to confirm the presence of intragenomic variability in the SSU rDNA of *B. variabilis*.
