# Supplementary material for: Can Abundance of Protists Be Inferred from Sequence Data: A Case Study of Foraminifera
Source: PLoS One. 2013 Feb 19;8(2):e56739. doi: 10.1371/journal.pone.0056739 (PMC3576339; doi:10.1371/journal.pone.0056739)
Supplement: Table S4 — SSU rDNA copy number estimation for Allogromia , Rosalina and Bolivina inferred from qPCR data and corresponding copy number factors relatively to Bolivina results, used for normalization of the sequence data. (DOC) [file pone.0056739.s004.doc]

Table S4: SSU rDNA copy number estimation for *Allogromia, Rosalina* and *Bolivina* inferred from qPCR data and corresponding copy number factors relatively to *Bolivina* results, used for normalization of the sequence data.

| species | qPCR 1 | Copy Number Factor (CNF) | qPCR 2 | Copy Number Factor (CNF) |
| --- | --- | --- | --- | --- |
| *Allogromia* | 41800 ± 8500 | 5.5 | 28300 ± 4300 | 6 |
| *Rosalina* | 11700 ± 4480 | 1.5 | 9900 ± 3100 | 2 |
| *Bolivina* | 7620 ± 4700 | 1 | 4450 ± 2700 | 1 |
