# Supplementary material for: Can Abundance of Protists Be Inferred from Sequence Data: A Case Study of Foraminifera
Source: PLoS One. 2013 Feb 19;8(2):e56739. doi: 10.1371/journal.pone.0056739 (PMC3576339; doi:10.1371/journal.pone.0056739)
Supplement: Table S5 — SSU rRNA expression level for Allogromia , Rosalina and Bolivina inferred from qPCR data and corresponding Expression Level Factors (ELF) relatively to Bolivina results, used for normalization of the sequence data. (DOC) [file pone.0056739.s005.doc]

Table S5: SSU rRNA expression level for *Allogromia, Rosalina* and *Bolivina* inferred from qPCR data and corresponding Expression Level Factors (ELF) relatively to *Bolivina* results, used for normalization of the sequence data.

| species | qPCR 1 | Expression Level Factor (ELF) |
| --- | --- | --- |
| *Allogromia* | 1.12*10^7^ ± 5.15*10^6^ | 1.5 |
| *Rosalina* | 3.81*10^7^ ± 1.1*10^7^ | 5.3 |
| *Bolivina* | 7.19*10^6^ ± 2.8*10^6^ | 1 |
