# Supplementary material for: Can Abundance of Protists Be Inferred from Sequence Data: A Case Study of Foraminifera
Source: PLoS One. 2013 Feb 19;8(2):e56739. doi: 10.1371/journal.pone.0056739 (PMC3576339; doi:10.1371/journal.pone.0056739)
Supplement: Table S6 — Number of rDNA sequences of Allogromia , Rosalina , Bolivina found after cloning and sequencing of five PCR products of the mixes of species (Replicate 1), RFLP analysis of five PCR products of the mixes of species (Replicates 2 and 3). (DOC) [file pone.0056739.s006.doc]

Table S6: Number of rDNA sequences of *Allogromia, Rosalina, Bolivina* found after cloning and sequencing of five PCR products of the mixes of species (Replicate 1), RFLP analysis of five PCR products of the mixes of species (Replicates 2 and 3).

|  |  | *Rosalina* | *Allogromia* | *Bolivina* | | Total |
| --- | --- | --- | --- | --- | --- | --- |
| Replicate 1 | Mix 3 | 1 | 78 | | 0 | 79 |
|  | Mix 10 | 17 | 74 | | 2 | 93 |
|  | Mix *Rosalina* | 67 | 24 | | 1 | 92 |
|  | Mix *Allogromia* | 1 | 93 | | 0 | 94 |
|  | Mix *Bolivina* | 6 | 32 | | 52 | 90 |
| Replicate 2 | Mix 3 | 19 | 133 | | 0 | 152 |
|  | Mix 10 | 32 | 98 | | 9 | 139 |
|  | Mix *Rosalina* | 132 | 36 | | 6 | 174 |
|  | Mix *Allogromia* | 3 | 165 | | 3 | 171 |
|  | Mix *Bolivina* | 9 | 50 | | 115 | 174 |
| Replicate 3 | Mix 3 | 10 | 176 | | 1 | 187 |
|  | Mix 10 | 32 | 151 | | 6 | 189 |
|  | Mix *Rosalina* | 128 | 58 | | 2 | 188 |
|  | Mix *Allogromia* | 0 | 187 | | 0 | 187 |
|  | Mix *Bolivina* | 9 | 68 | | 111 | 188 |
