# Supplementary material for: Can Abundance of Protists Be Inferred from Sequence Data: A Case Study of Foraminifera
Source: PLoS One. 2013 Feb 19;8(2):e56739. doi: 10.1371/journal.pone.0056739 (PMC3576339; doi:10.1371/journal.pone.0056739)
Supplement: Table S7 — Number of cDNA sequences of Allogromia , Rosalina , Bolivina found after cloning and sequencing of PCR products of the five mixes of species and RFLP analysis. (DOC) [file pone.0056739.s007.doc]

Table S7: Number of cDNA sequences of *Allogromia, Rosalina, Bolivina* found after cloning and sequencing of PCR products of the five mixes of species and RFLP analysis

|  | *Rosalina* | *Allogromia* | *Bolivina* | Total |
| --- | --- | --- | --- | --- |
| Mix 3 | 55 | 74 | 6 | 135 |
| Mix 10 | 103 | 11 | 17 | 131 |
| Mix *Rosalina* | 103 | 9 | 5 | 117 |
| Mix *Allogromia* | 42 | 75 | 2 | 119 |
| Mix *Bolivina* | 37 | 15 | 69 | 121 |
